# Constructing Epidemiologic Cohorts from Electronic Health Record Data

**DOI:** 10.3390/ijerph182413193

**Published:** 2021-12-14

**Authors:** Brent A. Williams

**Affiliations:** Geisinger Health System, Danville, PA 17821, USA; bawilliams2@geisinger.edu

**Keywords:** cohort studies, electronic health records, epidemiology, retrospective studies

## Abstract

In the United States, electronic health records (EHR) are increasingly being incorporated into healthcare organizations to document patient health and services rendered. EHRs serve as a vast repository of demographic, diagnostic, procedural, therapeutic, and laboratory test data generated during the routine provision of health care. The appeal of using EHR data for epidemiologic research is clear: EHRs generate large datasets on real-world patient populations in an easily retrievable form permitting the cost-efficient execution of epidemiologic studies on a wide array of topics. Constructing epidemiologic cohorts from EHR data involves as a defining feature the development of *data machinery*, which transforms raw EHR data into an epidemiologic dataset from which appropriate inference can be drawn. Though data machinery includes many features, the current report focuses on three aspects of machinery development of high salience to EHR-based epidemiology: (1) selecting study participants; (2) defining “baseline” and assembly of baseline characteristics; and (3) follow-up for future outcomes. For each, the defining features and unique challenges with respect to EHR-based epidemiology are discussed. An ongoing example illustrates key points. EHR-based epidemiology will become more prominent as EHR data sources continue to proliferate. Epidemiologists must continue to improve the methods of EHR-based epidemiology given the relevance of EHRs in today’s healthcare ecosystem.

## 1. Introduction

In the United States (US), electronic health records (EHR) are increasingly being incorporated into healthcare organizations in response to federal legislation financially incentivizing such organizations to implement EHR systems in a meaningful way or face penalties for non-adoption [1,2,3,4]. As of 2015, an estimated 85% of healthcare organizations in the US reported implementing an EHR system, and this percentage is expected to approach 100% [5]. Though the primary motivation for adopting EHRs is improved documentation of patient health and healthcare service provision with its expected benefits for healthcare quality, medical error reduction, proper reimbursement, and litigation protection, several secondary uses of EHR data show promise, including for the assessment of quality-improvement initiatives, tracking population health, and epidemiologic research [6,7,8,9,10,11]. EHRs serve as a vast repository of demographic, diagnostic, procedural, therapeutic, and laboratory test data generated during the routine provision of healthcare [1]. The appeal of using EHR data for epidemiology is clear: EHRs passively generate large datasets on real-world patient populations in easily retrievable form, allowing the cost-efficient and timely execution of epidemiologic studies on a broad array of topics [10,12,13,14]. Despite these conveniences, EHR-based epidemiology also entails a unique set of challenges that must be understood and resolved prior to its widespread application. 

### 1.1. Electronic Health Record Data as a Historical Cohort 

The most logical study design accommodated by EHR data is the historical (retrospective) cohort, though many of the issues discussed are relevant to other study types, including prospective studies [15,16,17,18]. EHR data are generated through the routine interactions of patients with healthcare organizations. When patients repeatedly patronize a particular healthcare organization, a longitudinal data stream develops whose value increases as time and data accrue [19,20]. Constructing epidemiologic cohorts from EHR data involves as a defining feature the development of *data machinery*, which transforms this raw EHR data stream into an epidemiologic dataset from which appropriate inference can be drawn. The remainder of this document focuses on three key aspects of data machinery development of high salience to constructing cohorts in EHR-based epidemiology: (1) selection of research participants; (2) defining “baseline” and assembly of baseline characteristics; and (3) follow-up for future outcomes. The defining features and unique challenges related to each are described. Furthermore, the unique features of EHR-based historical cohort studies are contrasted throughout with analogous features from prospective cohort studies (Table 1).

### 1.2. Ongoing Example: Diabetes and Heart Failure Hospitalization

Here, an ongoing example is introduced that will be referenced throughout, providing practical context to key points [21]. The historical cohort study includes 79,354 patients with type 2 diabetes mellitus (T2DM) followed longitudinally through a single healthcare organization’s EHR for a study endpoint of heart failure hospitalization (HFH). The primary goal of the study was to build a predictive model for HFH from a collection of candidate predictors derived from EHR data. The baseline date was an office visit with a known T2DM diagnosis. Baseline dates were assigned at least two years after the first EHR-documented encounter. Of note, T2DM could be either pre-existing or newly diagnosed at baseline. HFH was defined as a hospital admission with heart failure documented as the primary diagnosis.

## 2. Important Data Machinery Components for Historical Cohort Studies with EHRs

### 2.1. Selecting Research Participants

In a prospective cohort study, research participants typically consist of recruited volunteers meeting prespecified eligibility criteria willing to undergo the array of study procedures and agreeing to active follow-up at regular intervals, sometimes several years after enrollment. In an EHR-based retrospective study, eligible research participants are necessarily limited to individuals seeking services through healthcare organizations (i.e., patients). Importantly, some estimates suggest up to 50% of the US population has no contact with the healthcare industry in a given year [22]. This reality may have implications for generalizing findings from EHR-based epidemiologic studies to the broader target population [23]. Then, beyond inclusion criteria related to study-specific conditions, the predominant issue becomes selecting the appropriate research subset from a potentially vast EHR patient database.

Individuals interact with healthcare organizations in different ways, and these differences invariably impact the availability and quality of information drawn from an EHR [6]. Furthermore, in the US, patients often seek care through multiple healthcare organizations, and data linkage between organizations is often not possible, which also hinders information quality [1,24,25,26,27,28]. Information quality in the context of EHR-based studies has two overlapping yet distinct attributes: completeness and correctness. Data (in)correctness—e.g., an erroneously documented diagnostic code, quantitative errors—is a legitimate concern in EHR-based research, but unfortunately, is often unidentifiable and largely uncorrectable by researchers in a retrospective context. Data completeness, on the other hand, is under greater researcher control in that criteria can be applied to enhance completeness in the research subjects ultimately chosen. Unfortunately, data completeness—that is, the capacity of EHR data to fully characterize an individual’s medical state—is a nebulous construct when appraising retrospective EHR data where true “completeness” is ill-defined [25,29]. However, completeness clearly has a positive association with the frequency of interaction with a healthcare organization (i.e., more interaction implies greater completeness) and types of interaction (e.g., primary care visits typically generate more information than specialty visits). Thus, there is a rationale for applying some sort of “information completeness” metric when selecting research participants from an EHR database—and only including patients exceeding a given threshold—but such criteria can be difficult to define in an appropriately objective manner and are largely untestable [1,7,29]. Furthermore, including research participants based on (suspected) data completeness will inevitably over-select less-healthy patients given their greater need for healthcare services [29,30,31,32,33]. Indeed, a significant ongoing analytical challenge in EHR-based epidemiology is accounting for such differential data completeness [34].

**Figure 1:**
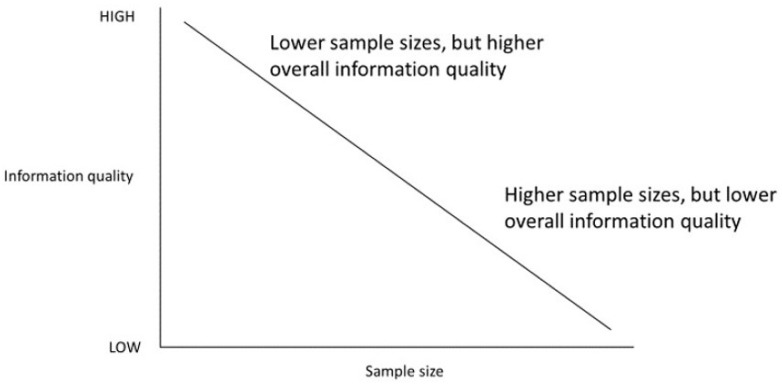
The information quality spectrum of electronic health record data. Patients within an electronic health record database have highly variable information quality that is dependent on multiple factors such as frequency and types of interactions with healthcare organizations. Moving down the information quality spectrum allows more patients to be included in epidemiologic studies, but at the expense of information quality.

Toward achieving the data-completeness objective, one logical starting point for setting study inclusion criteria when warranted by a study’s objectives includes receipt of primary care services through the healthcare organization [7]. The nature of primary care visits typically allows a greater proportion of a patient’s medical information to be captured, and primary care often serves as a conduit to more specialized services, which would often be funneled through the same healthcare organization when needed (provided the institution offers such services). Thoughtful consideration of study inclusion criteria is important as only a fraction of patients in an EHR database may have acceptable information quality and tradeoffs must be made between information quality and sample size in that permitting more patients into a study is only accomplished at the expense of reduced information quality among the additional patients (Figure 1). In the T2DM-HFH example, the base population from which T2DM patients were drawn consisted of approximately 500,000 patients who received primary care services through the study institution for at least two years since the first EHR-documented encounter [21]. This research subset represented just one-third of all patients in the entire EHR database. 

### 2.2. Defining “Baseline” and Assembly of Baseline Characteristics

In prospective cohort studies, a clear study start date is predefined; for instance, the date of diagnosis of a certain condition, the date a specific procedure was performed, or, in a study enrolling generally healthy volunteers, the date informed consent was obtained. This start date is typically referred to as the “baseline date” (or simply “baseline”) and serves as the starting point for the follow-up of study outcomes. A typical prospective study measures an extensive collection of baseline characteristics through various means such as questionnaires, blood draws, and perhaps novel measurement devices such as imaging. Notably, prospective studies by design measure baseline characteristics in a standardized, protocol-specified manner among all study participants.

**Figure 2:**
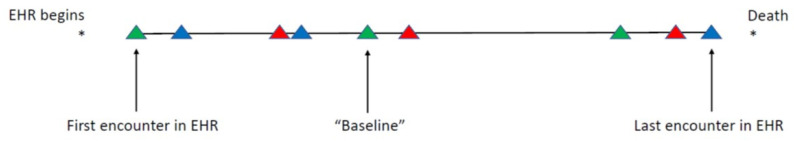
An individual patient’s electronically documented journey through a healthcare organization depicted as a timeline, bookended by the first and last EHR-documented encounters, with multiple, variably spaced, and qualitatively different types of encounters between. Colored triangles represent different encounter types.

In an EHR-based retrospective cohort study, each patient’s electronically documented journey through a healthcare organization can be depicted as a timeline, bookended by the first and last EHR-documented encounters with multiple, variably spaced, and qualitatively different types of encounters between (Figure 2). Here, an *encounter* is broadly defined as any professional contact between a patient and healthcare organization, including primary care, specialty care, laboratory testing, emergency department visits, hospital admissions, and others [1]. Even less-direct patient–provider contact such as telephone calls and email can be considered encounters when they provide useful information for a research study. Notably, a healthcare organization may offer only limited types of encounters (e.g., a standalone hospital), which may be restrictive for research. The baseline date for any EHR-based retrospective study can fall on or anywhere between the first and last encounters, but certain considerations prevail. First, baseline dates should be assigned in proximity to the first EHR-documented encounter (when sensible) so that post-baseline follow-up time is maximized. However, any single encounter seldom provides sufficient detail for adequate baseline characteristic assessment, making the first encounter a generally unappealing baseline date [4]. Allowing more time and encounters to accrue prior to the baseline date assignment permits a more comprehensive baseline assessment but at the expense of reduced sample size and follow-up duration [35,36,37,38,39]. Indeed, it is common for retrospective studies using electronic data sources to require at least six months but typically two years or more of pre-baseline encounter information [35,36,37,38,39]. By the nature of the data-generating process, sicker patients with several existing medical conditions typically need more encounters to provide a complete medical picture, while heathier patients require fewer [29]. 

#### 2.2.1. EHR Data: What Is Available

The data elements eligible for consideration in any EHR-based epidemiologic study are those documented during the usual course of healthcare service provision and can be generally grouped into demographics, vital signs, diagnoses, procedures, medications, and laboratory tests [1,2,40]. The quantitative (how much) and qualitative (how good) attributes of EHR data undoubtedly rely on default measurement processes implemented within the clinical enterprise and documentation tactics of individual healthcare providers [6,18,34]. Physicians spend an estimated 20% of their professional time documenting clinical encounters, and though incentives exist to be exhaustive in documentation (to maximize reimbursement) while not over-documenting (to avoid fraud), it is impossible to retrospectively determine how well these principles were adhered to in practice [3,4,5]. Misclassification of categorical characteristics, the measurement error of continuous characteristics, and missing data are concerns in every epidemiologic study—concerns that are magnified in EHR-based research by the nature of the data-generating process [25,32,41,42]. Unfortunately, from a researcher’s perspective, there are no simple solutions to correcting these data limitations at their source, but actions can be taken to minimize their impact.

#### 2.2.2. Opportunity for Information

**Figure 3:**
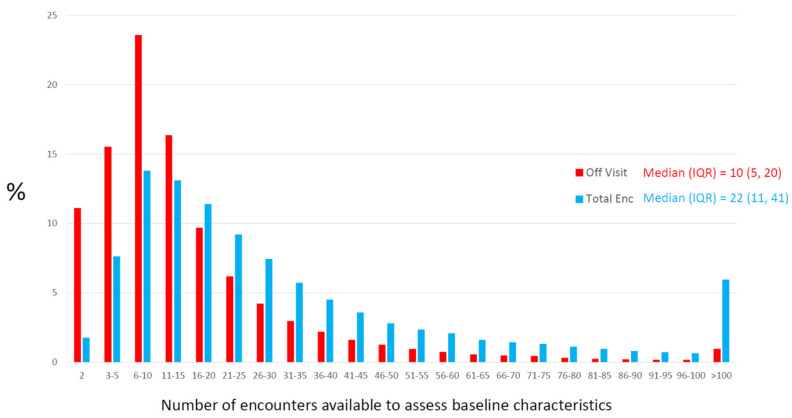
Opportunity for Information—diabetes and heart failure hospitalization example. Relative frequency histograms of the number of encounters used to determine baseline characteristics for all encounters (in blue) and office visits only (in red).

When determining baseline characteristics in an EHR-based retrospective study, the baseline date serves as the reference point by which study variables are assigned a present/absent status for dichotomous variables or a numerical value for continuous variables. Baseline information is assembled from encounters occurring on or prior to the baseline date, and when appropriate, from encounters occurring shortly following baseline (e.g., 90 days). In sharp contrast to the prospective study environment, an EHR-based retrospective study simply cannot standardize the process of organizing baseline characteristics in any completely acceptable way. In fact, when considering all possible permutations of the quantitative (number of encounters) and qualitative (e.g., primary care, ED visits) ways patients could interact with healthcare organizations, it is more likely that no two patients will have had their baseline characteristics obtained in the same way. Here, a new construct is introduced—*Opportunity for Information* (OFI)—to describe the collection of encounters that could provide usable baseline information (Table 2). Re-expressing the initially stated concern in terms of the OFI, it is likely that the OFI has substantial inter-patient variability in any EHR-based research study. OFI can be quantified in terms of time, or the number of encounters from the first EHR-documented encounter to the baseline encounter. An OFI measure based on certain encounter types (e.g., the number of primary care visits) may also be considered. In the T2DM-HFH example, the baseline date was assigned according to the first office visit after two years had elapsed since the first EHR-documented encounter and a primary care visit had occurred. Using this baseline definition, the mean (SD) OFI time was 4.6 (3.3) years, with a range of 2.0 to 14.8 years. This wide variation in OFI time partially reflects the inclusion of both pre-existing (at baseline) and newly diagnosed T2DM in the study; as expected, pre-existing T2DM had a much shorter mean OFI time compared to new diagnoses (3.5 vs. 6.7 years). When describing OFI variability in terms of the encounter frequency, again, a wide dispersion in OFI is observed (Figure 3). The median (IQR) number of encounters available for determining baseline characteristics was 22 (11, 41), and the range was 2 to 1437 (in Figure 3, the number of office visits is shown separately as the most informative encounter type).

As the above example reveals, the amount of eligible information for assembling baseline characteristics can be highly variable across study patients, even among a collection of patients all adhering to a minimal set of information criteria. The primary concern associated with inter-patient OFI variability is the dependency of the documented presence of certain baseline characteristics to the OFI, implying an association between OFI and the misclassification rate for certain binary characteristics [35,37]. In particular, EHR documentation of intermittent, transient, and/or more subjectively determined characteristics (e.g., depression, shortness of breath) is positively associated with the OFI, but permanent, common, and more objective features (e.g., hypertension) less so [43,44]. Inevitably, lower OFI leads to the greater false-negative classification of baseline features relative to higher OFI [45]. Unquestionably, valid identification of pre-existing medical conditions at baseline will be partially dependent on the OFI in that study patients with greater OFI will logically have more opportunity for documentation of a medical condition [38,44]. A specific problem arises when the association between two OFI-dependent variables is of interest, as an artificially inflated association can be induced [43,44]. The phenomenon acts quantitatively similar to usual confounding, and under the appropriate conditions, can be controlled with the usual confounding-correction tactics (by an OFI metric) [43,44]. However, these tactics can be challenging to implement in practice, particularly with several covariates, some OFI-dependent, but others not [43,44].

**Figure 4:**
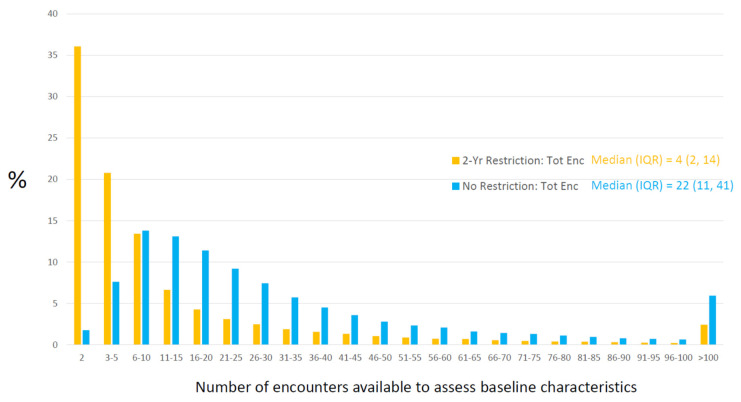
Opportunity for Information—diabetes and heart failure hospitalization example: the effect of applying a two-year, pre-baseline time restriction. Relative frequency histograms of the number of total encounters used to determine baseline characteristics with no pre-baseline time restriction (in blue) and restricting to encounters occurring within two years of baseline (in orange).

In an attempt to control for inter-patient variability in OFI and standardize the baseline information-gathering process, many studies using electronic data sources (EHRs, insurance claims) have applied a fixed, pre-baseline time interval for baseline assessment, which ignores encounter information prior to the interval. The tactic will standardize OFI time (SD = 0), but inter-patient variability in encounter-based OFI metrics will ultimately persist. Figure 4 shows the reduction in the number of encounters considered for baseline assessment when applying a fixed, 2-year pre-baseline time restriction in the T2DM-HFH example (mean OFI time decreases from 4.6 to 2.0 years). The median number of total encounters considered drops from 22 to 4, and the restriction results in about one-third of patients using only two encounters to determine baseline characteristics (Figure 4). This attempt at standardization also creates a new problem by increasing the rate of false-negative misclassification [46]. Indeed, the strategy knowingly changes (presumed) correct information into incorrect information. Table 3 shows the decrease in the prevalence of a subset of baseline characteristics from the T2DM-HFH example when applying a 2-year time restriction. As the table suggests, applying a pre-baseline time restriction may have little impact on the interpretation of aggregate numbers, yet comparing counts with and without the restriction reveals the extent of misclassification induced. Though no method is without limitation, a strategy incorporating all available pre-baseline encounters seems preferable [46].

#### 2.2.3. Creating Rules for the 99%

The assembly of baseline characteristics derived from the vast array of elements available within EHRs involves *creating rules for the 99%*—an informal expression implying that imperfect rules must be implemented that work well for the majority but rarely universally. The counterpoint is that it is often possible to find scenarios where strict application of a proposed rule provides incorrect information; however, changing the rule to accommodate the scenario improperly changes correct information for many more study subjects and could thus be counterproductive. For instance, in T2DM, requiring documentation of an elevated hemoglobin A1c as part of a study’s diagnostic criteria could misclassify patients deemed diabetic by diagnosis codes only (but with missing A1c). Rule creation for qualitative variables usually involves observing the appropriate structured data elements at encounters within specified time intervals, perhaps with additional criteria based on frequency (e.g., requiring >1 occasion of a code), temporal proximity (e.g., requiring <1 year between separate code occasions), and/or context (e.g., primary diagnoses given precedence over secondary) [7,47,48,49,50,51,52]. Ultimately, imperfect rules must be implemented, and researchers must accept a tolerance for this noise. Indeed, a reliance on rules is mandatory as EHR data sets are typically too large for exhaustive manual validation of any data element. Fortunately, direct observation of electronic medical charts permits scrutiny of EHR-based rules, allowing researchers to “pull back the curtain” to uncover and correct suboptimal rules—a feature not available with most insurance-claims-based studies.

#### 2.2.4. Hidden Missingness

By the nature of clinical documentation processes, EHR-based rules for binary characteristics are largely restricted to positive affirmations for defining disease “presence” (i.e., observing a documented code)*,* and the absence of positive affirmations (i.e., not observing a code) for defining disease “absence” (see phrase “looking for yes”—Table 2) [53]. That is, structured EHR data rarely contain negative affirmations—documentation that a certain disease was sought but not found, which would lend greater credence to its true absence. As such, EHR-based studies have an inherent inability to differentiate “no disease” from “missing disease status”—the former defined by the clinical situation where a specific disease was sought but not found, and the latter defined by a disease not sought in any clinical context [26,53]. As a consequence, given the dependence of certain diagnoses being documented on the OFI, EHR studies will ultimately possess a degree of *hidden missingness*—qualitative diagnoses labeled “not present” according to rule criteria that were simply not investigated under usual clinical circumstances. The extent of hidden missingness will be related to information quality (and the OFI) and creates a misclassification problem. Two related concepts introduced here are *weak no* and *strong no*—the former describing disease-absence labels based on weak information, and the latter on strong information.

#### 2.2.5. Quantitative Data: Measurement Error and Missing Data

The quantitative data typically available within EHRs (e.g., vital signs, laboratory test results) are a commonly cited strength of EHR data relative to other electronic data sources such as insurance claims. The process of assigning a single numerical value for a quantitative data element to a baseline date should involve a hierarchical, temporal prioritization which first favors a value measured on the baseline date, then the value measured closest in time prior to baseline (perhaps with some limit on how far back in time is allowable), and then finally, when appropriate, the value measured closest in time after baseline (with a definite limit on how far forward in time is allowable) [54]. The setting in which the measurement was taken may also warrant consideration (i.e., outpatient vs. inpatient). Inevitably, measurement errors and missing data will abound [12]. Random variation of quantitative measurements from an EHR is often greater than analogous measurements taken under a standardized, prospective research protocol, and is largely uncorrectable (e.g., blood pressure [55]). Furthermore, missing data in an EHR are seldom missing at random [13,56,57]. For instance, in one study, the measurement of certain quantitative health indicators varied by demographic characteristics, the extent of chronic disease, and treatment status [56]. Oftentimes, missing data imply better health in ways that are undocumented [58]. The tenuousness of the missing-at-random assumption complicates the application of popular imputation techniques [58].

### 2.3. Follow-Up for Future Outcomes

Outcome tracking in prospective studies typically involves regularly timed, standardized, and complete follow-up assessments of all study participants with the adjudication of suspected study endpoints. In an EHR-based historical cohort study, outcome-tracking is inherently passive. The outcome-tracking time interval starts on the baseline date and continues through the last EHR-documented encounter or death [20]. The identification of outcome occurrences is also subject to the *creating rules for the 99%* principle, as described above. Additionally, again, in EHR studies, direct access to electronic records allows for the adjudication of study outcomes and/or assessment and possibly alteration of the outcome-defining rule.

Ideally, all study outcomes among study participants occurring within the follow-up interval would be identified, though the ability of any healthcare organization’s EHR data to accomplish exhaustive outcome detection depends on the extent to which study outcomes manifest at the study institution. In the US, certain features of the healthcare-delivery ecosystem might impede exhaustive outcome detection through any single healthcare organization. First, patients often receive healthcare services through multiple organizations, and these organizations seldom share data, especially for research [19,24,25,26,28,45,59]. Second, many healthcare organizations only provide a limited set of healthcare services, so certain events may never manifest at these institutions, leading to event undercounting [60,61,62,63]. In the context of an EHR-based retrospective study, a simplifying assumption is often made that study patients are validly tracked for study outcomes within the entire follow-up period, and in contrast, that the absence of an EHR-documented study outcome equates to no event occurrence [35,64]. These assumptions are made as it is difficult or frequently impossible to differentiate from a single organization’s EHR non-occurrence of study outcomes from outcomes occurring at an external organization. These attributes of EHR-based follow-up contrast with analogous retrospective studies using insurance-claims data, where any salient health event occurring within a follow-up interval is assumed to be identified with near certainty. The issue of missed events is especially relevant when acute, life-threatening, but ultimately non-fatal events are among the outcomes being tracked (e.g., myocardial infarction).

## 3. Conclusions

Drawing correct inference and estimating minimally biased effects from EHR-based retrospective studies greatly depends on identifying the most informative patients from an EHR database without compromising the generalizability. The various ways and intensities by which individuals utilize healthcare services suggest a substantial fraction of patients in an EHR system will have data shortcomings (i.e., do not meet a minimal data-completeness standard) and should not be included in research studies. Focusing on patient selection with an eye toward maximizing data completeness is a logical strategy but defies a completely objective definition and will tend to over-select a less-healthy patient population. Requiring receipt of primary care services through the EHR-bearing organization within the study’s inclusion criteria should impart greater confidence in a more complete ascertainment of baseline characteristics and future study outcomes, but the overall spectrum of services provided by the organization and the extent to which patrons use that spectrum are also vital from a data-completeness perspective. Other attributes such as a healthcare organization’s reputation and the number of competing healthcare organizations in the geographic area can also impact data completeness. In most cases, some data integrity must be compromised to take full advantage of the large patient populations and unprecedented longitudinal detail which characterize mature EHR databases. Hidden missingness is unavoidable and impossible to quantify, yet precautions can be taken to minimize its impact through proper patient selection and rule creation. Unfortunately, it is easy to perform an EHR study that generates believable results yet is fraught with preventable misclassification and missing data. Results generated from EHR studies can have an aura of credibility because of highly precise results derived from large sample sizes, yet they can be severely biased [65]. Despite these limitations, EHR-based retrospective studies will likely become more prominent as EHR databases proliferate. Epidemiologists must continue to improve the methods of EHR-based epidemiology, given the relevance of EHRs in today’s healthcare environment.

## Figures and Tables

**Table 1:** Comparison of study design attributes in prospective vs. retrospective studies using electronic health records.

	Prospective Study	Retrospective Study with EHR
Selecting Research Participants	Inclusion and exclusion criteria prospectively applied Volunteers willing to undergo study procedures and be actively followed	Inclusion and exclusion criteria applied post hoc Patrons of a healthcare organization Preferably exceed some minimal information threshold
Baseline	Often date of some salient health event such as diagnosis of a condition, procedure performed, or date informed consent provided among generally healthy volunteers	Any time on or between first and last EHR-documented encounters Preferably on the date of an actual encounter Preferably not first encounter
Assembling Baseline Characteristics	At baseline, measured by questionnaires, blood tests, imaging, and other measurement instruments in a standardized manner among all study participants	Only considers data elements collected during usual care Uses encounters occurring on or before the specified baseline Qualitative characteristics determined by rules for the 99%
Follow-up for Future Outcomes	Active, standardized follow-up at regular intervals in all study participants Adjudication of claimed outcomes	Passive follow-up Only study outcomes documented at study institution are identified Rules for the 99% apply

**Table 2:** Definitions of terms and phrases.

Term or Phrase	Definition
Encounter	Any professional contact between a patient and healthcare organization, including primary care, specialty care, laboratory testing, emergency department visits, hospital admissions, etc.
Opportunity for Information	The collection of pre-baseline encounters that could provide usable research information. Can be expressed in units of time (days from first encounter to baseline encounter) or as number of encounters (between first and baseline encounters).
Creating Rules for the 99%	When assembling baseline characteristics for an EHR-based retrospective study, rules must be created for determining presence/absence of qualitative characteristics and values for quantitative characteristics. This informal expression implies that imperfect rules must be implemented that work well for the majority but rarely universally.
Looking for Yes	An expression applied when determining the presence/absence of a binary characteristic, denoting how rules typically only look for positive affirmations of the characteristic and rarely negative affirmations.
Hidden Missingness	A phrase describing the scenario where a qualitative condition (e.g., diagnosis) is labeled “absent” but was never queried nor investigated in clinical practice. Thus, the condition’s true status as present/absent is actually undetermined despite being labeled “absent”.
Weak No	A scenario where a qualitative condition (e.g., a diagnosis) is labeled absent based on weak information.
Strong No	A scenario where a qualitative condition (e.g., a diagnosis) is labeled absent based on strong information.

**Table 3:** Loss of information when restricting pre-baseline time intervals for assessment of baseline characteristics.

Baseline Characteristic	No Restriction	2-Year Restriction
Hypertension	71% (*n* = 56,653)	67% (*n* = 53,350)
High cholesterol	69% (*n* = 54,652)	64% (*n* = 51,003)
Coronary bypass surgery	7% (*n* = 5293)	6% (*n* = 4673)
Heart failure	11% (*n* = 9026)	10% (*n* = 8170)
Acute myocardial infarction	8% (*n* = 6516)	7% (*n* = 5362)
Chest pain	22% (*n* = 17,179)	15% (*n* = 12,141)
Shortness of breath	16% (*n* = 12,993)	12% (*n* = 9784)
Depression	25% (*n* = 19,812)	21% (*n* = 16,901)

Numbers in table cells are percentage (number) of patients with a history of the characteristic. Denominator size is 79,354.

## Data Availability

Not applicable.
